# Expression of Immunoglobulin G4 in Eosinophilic Esophagitis

**DOI:** 10.3390/jcm13082175

**Published:** 2024-04-10

**Authors:** See-Young Lee, Ji-Hae Nahm, Min-Jae Kim, Yuna Kim, Jie-Hyun Kim, Young-Hoon Youn, Hyojin Park

**Affiliations:** 1 Division of Gastroenterology, Department of Internal Medicine, Gangnam Severance Hospital, Yonsei University College of Medicine, Seoul 06229, Republic of Korea; seeyoung87@yuhs.ac (S.-Y.L.); kmj0630@yuhs.ac (M.-J.K.); sadts@yuhs.ac (Y.K.); otilia94@yuhs.ac (J.-H.K.); dryoun@yuhs.ac (Y.-H.Y.); 2 Department of Pathology, Gangnam Severance Hospital, Yonsei University College of Medicine, Seoul 06229, Republic of Korea; nam2169@yuhs.ac

**Keywords:** eosinophilic esophagitis, immunoglobulin G, EoE endoscopic phenotype

## Abstract

**Background**: Eosinophilic esophagitis (EoE) is a disease that has been subcategorized into two endoscopic phenotypes: inflammatory and fibrostenotic. Moreover, studies have shown a link between EoE and immunoglobulin G4 (IgG4), a subclass of the immunoglobulin G (IgG) antibody. In this study, we aimed to evaluate the relationship between histologic IgG4 expression and endoscopic phenotypes in patients with EoE. **Methods**: This case-control study included patients diagnosed with EoE (*n* = 19) and patients with non-obstructive dysphagia without abnormal findings as controls (NOD; *n* = 12). The EoE group was further divided into three subgroups based on endoscopic phenotype: inflammatory, fibrostenotic, or combined. Retrospective examination of endoscopic findings and pathological slides was performed to analyze IgG4 staining. **Results**: Histological analysis revealed a significant difference in IgG4 cell count (15.00 vs. 0.58, *p* = 0.003) and eosinophil cell count (84.67 vs. 0.08, *p* < 0.001) between the EoE and NOD groups. Symptom manifestation and blood test results were similar across all three endoscopic EoE phenotypes. However, histological analysis revealed a significant difference in IgG4 cell count between the inflammatory, fibrostenotic, and combined phenotypes (4.13 vs. 17.6 vs. 59.7, *p* = 0.030). **Conclusions**: IgG4 expression was higher in EoE patients than in those with NOD, the highest being in the combined phenotype subgroup. These findings emphasize the important role of endoscopic and histological examination in diagnosing EoE and the need for further research in this area.

## 1. Introduction

Eosinophilic esophagitis (EoE) is a chronic immune-mediated disorder of the esophagus that is characterized by eosinophilic inflammation [1,2]. The prevalence and incidence of EoE have increased since it was first described in 1978 [3]. Currently, EoE is the second-most prevalent disease of the esophagus after gastroesophageal reflux disease [4]. The condition is increasingly recognized as a major cause of dysphagia, food impaction, and gastroesophageal reflux disease-like symptoms in children and adults. Although the pathogenesis of EoE remains poorly understood, it is believed to be multifactorial, involving both genetic and environmental factors. Esophageal infiltration of eosinophils is the most important finding in EoE diagnosis. However, esophageal infiltration can also be observed in reflux esophagitis, esophageal infection, and drug-induced esophagitis [5].

Recent studies have shown the association between EoE and immunoglobulin G4 (IgG4), a subclass of the immunoglobulin G (IgG) antibody that is associated with various immune-mediated diseases [6,7,8,9]. IgG4 is involved in immune tolerance, and its increased expression is associated with several immune-mediated diseases, including autoimmune pancreatitis, sclerosing cholangitis, and interstitial lung disease [10]. Increased IgG4 levels are detected in the serum and esophageal tissue of patients with EoE, suggesting its potential role in disease pathogenesis [8]. IgG4-positive plasma cells are also present in the esophageal mucosa of patients with EoE, particularly in the areas of active inflammation [11].

Based on the visual appearance of the esophageal mucosa during endoscopy, EoE can be broadly divided into inflammatory and fibrostenotic phenotypes. Edema, furrows, and exudate are mainly observed in the inflammatory phenotype, whereas rings and strictures are mainly observed in the fibrostenotic phenotype. These phenotypes are associated with different clinical presentations and treatment responses. For example, furrows are characterized by linear grooves or gaps in the esophageal mucosa and often associated with dysphagia and strictures, whereas rings are circular constrictions that can cause food impaction [12].

IgG4-related diseases are characterized by chronic inflammation and fibrosis due to elevated serum IgG4 levels and an immune response from IgG4-positive plasma cells, which leads to fibrosis in surrounding tissues [13]. However, the impact of EoE on the esophagus has not been as thoroughly understood as its effects on other organs. Nevertheless, case reports and studies have indicated possible involvement [14]. A shared characteristic of the clinical progression in both IgG4-related disease and EoE is their propensity to induce tissue fibrosis. However, the connection between esophageal lesions observed in patients with EoE and this fibrotic process remains unknown [15,16,17]. Thus, given the potentially different roles of endoscopic phenotypes and emerging evidence linking IgG4 to EoE, understanding how IgG4 expression varies depending on the endoscopic phenotype of EoE is important.

Therefore, in this study, we aimed to assess the relationship between IgG4 expression and the endoscopic phenotype of EoE to provide insight into the underlying mechanisms and potential treatment strategies for this challenging condition. Specifically, we aimed to evaluate IgG4 levels in the esophageal tissue of patients with different endoscopic phenotypes of EoE and investigate potential correlations with clinical presentations and treatment outcomes. Additionally, we aimed to evaluate the distribution and density of IgG4-positive plasma cells in the esophageal mucosa of patients with EoE. We believe the results of this study will contribute to our understanding of the pathogenesis of EoE and have implications for its diagnosis and treatment.

## 2. Materials and Methods

### 2.1. Study Design and Patient Selection

**Figure 1:**
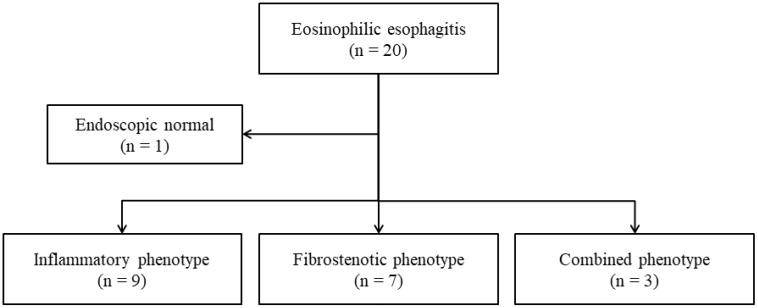
Flowchart of the study participants. Patients complaining of dysphagia were classified into the EoE or NOD groups based on biopsy results. Patients with EoE were divided into inflammatory, combined, or fibrostenotic phenotypes based on their endoscopy results. In this process, one patient with EoE was excluded from phenotype classification owing to normal endoscopy findings. EoE, eosinophilic esophagitis; NOD, non-obstructive dysphagia.

The study protocol was approved by the Institutional Review Board of Gangnam Severance Hospital, Republic of Korea (IRB number 2022-0540-002). This case-control study included patients diagnosed with EoE (*n* = 19) and with non-obstructive dysphagia (NOD; *n* = 12). The medical records of patients presenting with dysphagia at Gangnam Severance Hospital, Yonsei University between March 2010 and March 2021 were reviewed. Baseline characteristics including age, sex, patient symptoms (i.e., dysphagia, heartburn, dyspepsia, regurgitation, food impression), blood test results, endoscopic findings at the time of initial diagnosis (i.e., exudation, depression, crêpe paper-like mucosa, rings, strictures), and Charlson Comorbidity Index (CCI) were recorded. During their initial visit, patients underwent diagnostic laboratory tests, including a complete blood count (CBC) and evaluation of immune biomarkers. The analysis specifically targeted immunoglobulin levels (IgG, IgG4, and IgE) to ascertain the immunological profile of the subjects. These examinations were performed following the hospital’s standardized diagnostic laboratory protocols to ensure consistent and accurate results. Patients were diagnosed with EoE if their esophageal histology showed more than 15 eosinophils per high-power field (HPF) according to the guidelines [1]. Patients with NOD were defined as those who complained of symptoms such as dysphagia but had normal findings on endoscopy, esophagography, esophageal manometry, and histological examination. The exclusion criteria for the study were as follows: the presence of any diseases or conditions that could potentially interfere with or confound the study outcomes, active infections such as *Helicobacter pylori* or esophageal varices, any evidence of immunosuppression, and recent administration of systemic immunosuppressive or immunomodulatory medications. Patients in the EoE and NOD groups were age- and sex-matched as closely as possible and had no significant past medical history. Patients in the EoE group were further classified into inflammatory (*n* = 9), fibrostenotic (*n* = 7), or combined (*n* = 3) phenotypes based on their endoscopic examination results. One patient diagnosed with EoE was excluded from the phenotypic classification owing to normal endoscopic examination results (Figure 1).

### 2.2. Histological Analysis

**Figure 2:**
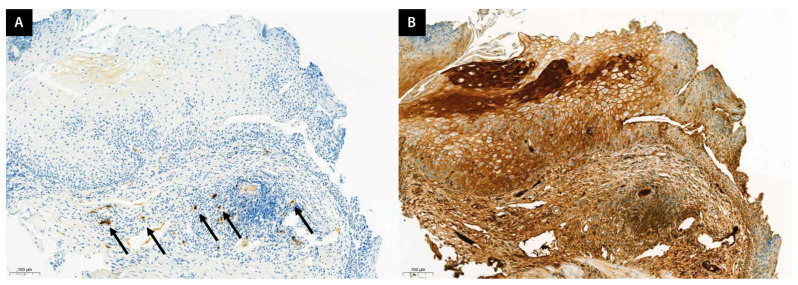
Representative histological analysis of IgG4 and IgG. (**A**) Example of IgG4 immunostaining shows IgG4 plasma cells (arrow) in the subepithelial area. (**B**) The ratio of IgG4 to IgG is calculated by counting the number of IgG plasma cells at the same location (9/228 × 100 = 3.94%). Ig, immunoglobulin.

Esophageal biopsy specimens were initially preserved in 10% neutral buffered formalin for a full day and subsequently rinsed in distilled water. The specimens were sequentially dehydrated through varying concentrations of alcohol, followed by clarification in xylene before being embedded in paraffin. Slices of 5 µm thickness were prepared using a microtome and then stained using hematoxylin and eosin (H&E) as well as immunohistochemistry (IHC) for comprehensive histological examination. An independent pathologist reviewed the slides. Eosinophil counts were meticulously documented from the H&E stained images to assist in EoE diagnosis. The presence of IgG4-producing plasma cells was identified through IgG4 IHC staining, and the IgG4 to IgG ratio was determined by quantifying IgG4-positive cells in clustered regions (Figure 2).

**Figure 3:**
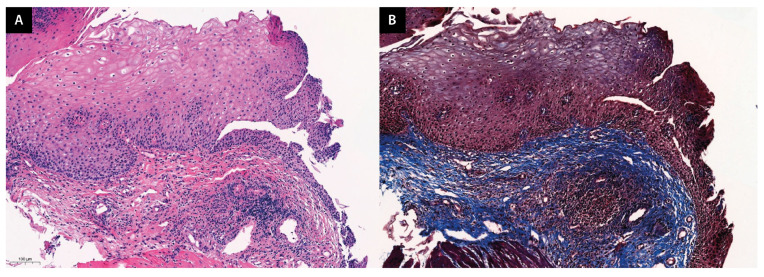
Representative histological analysis of esophageal tissue. (**A**) Hematoxylin and eosin-stained slide. (**B**) TRC-stained slide. TRC staining is used to visualize collagen-driven fibrosis, which appears blue in the image. The presence of blue bundles in the TRC-stained slide indicates advanced fibrosis (note that storiform fibrosis, which is characteristic of IgG4-related disease, is not observed in this patient). TRC, Masson’s trichrome.

Further, we used Masson’s trichrome (TRC) staining to examine the association of IgG4 with fibro-inflammatory disease [18] and confirm whether the collagen layer displayed fibrosis (Figure 3).

### 2.3. Endoscopic Analysis

**Figure 4:**
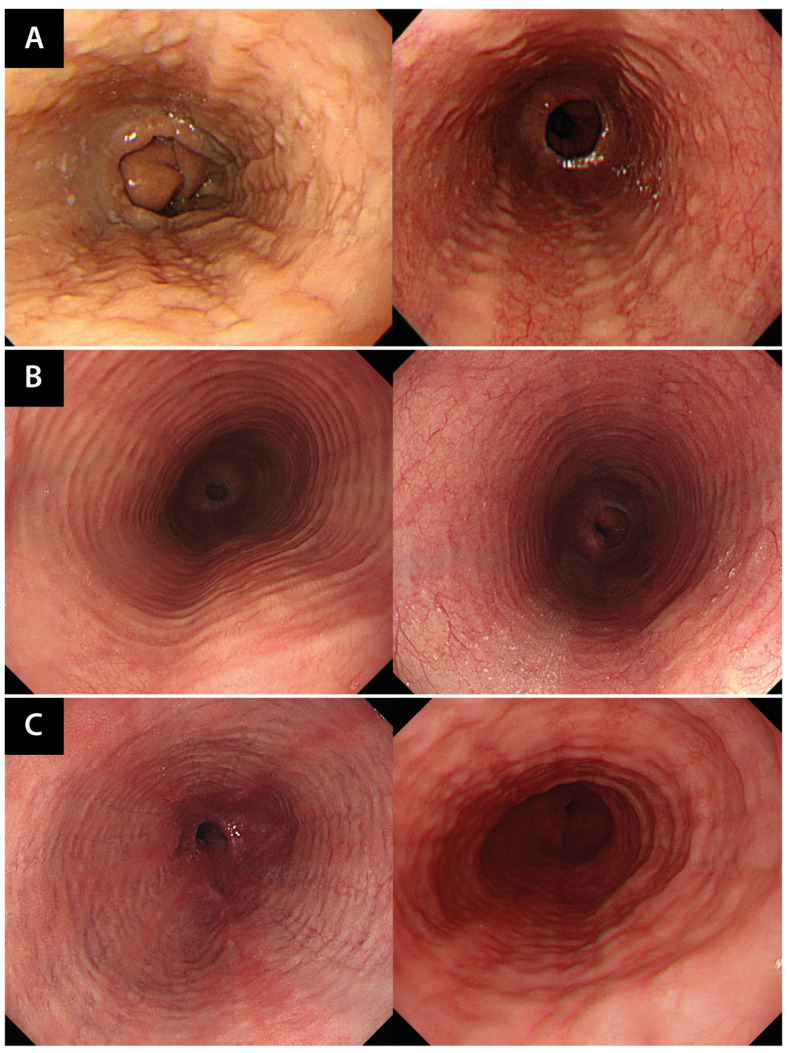
Representative endoscopic analysis of EoE. (**A**) Inflammatory phenotype. Left image from pre-treatment endoscopy reflects an EREFS of 3 (exudate score of 1 and furrow score of 2). Right image from post-treatment endoscopy reflects an EREFS of 1 (exudate score of 0 and furrow score of 1). The ΔEREFS for this patient is 2. (**B**) Fibrostenotic phenotype. Left image from pre-treatment endoscopy reflects an EREFS of 2 (ring score of 2). Right image from post-treatment endoscopy reflects an EREFS of 1 (ring score of 1). The ΔEREFS for this patient is 1. (**C**) Combined phenotype. Left image from pre-treatment endoscopy reflects an EREFS of 3 (furrow score of 1 and ring score of 2). Right image from post-treatment endoscopy reflects an EREFS of 3 (furrow score of 1 and a ring score of 2). The ΔEREFS for this patient is 0. EoE, eosinophilic esophagitis; EREFS, Endoscopic Reference Score; ΔEREFS, change in EREFS.

Endoscopic analysis was performed using standard endoscopes (GIF-Q260J, GIF-H260, GIF-H290, Olympus Medical Systems, Inc., Tokyo, Japan) to identify the presence of edema, exudate, furrows, rings, and strictures (Figure 4). We used the Endoscopic Reference Score (EREFS), which is an established endoscopic tool for assessing the presence and severity of EoE [19], to determine the correlation between TRC staining and the likelihood of endoscopic reversibility. Additionally, follow-up endoscopy was conducted 3 months after treatment initiation to monitor changes in EREFS (ΔEREFS) over time to assess the dynamic nature of the disease.

Based on the endoscopic features, the EoE patients were categorized into three phenotypes. The inflammatory phenotype was characterized by edema, exudate, and furrows. The fibrostenotic phenotype was characterized by rings and strictures. The combined phenotype exhibited both types of endoscopic feature.

### 2.4. Statistical Analysis

Demographic characteristics between the groups were compared using Mann–Whitney U tests for continuous variables and Fischer’s exact tests for proportions and categorical variables. Tissue IgG cell counts were analyzed using Mann–Whitney U tests for between-group comparisons and one-way analysis of variance (ANOVA) for within-group comparisons. Associations between tissue IgG4 cell counts and endoscopic features were examined using Wilcoxon signed-rank tests. Statistical significance was set at *p* < 0.05. All statistical analyses were performed using SPSS version 26.0 (IBM Corp., Armonk, NY, USA).

## 3. Results

### 3.1. Baseline Characteristics of EoE and NOD Groups

Overall, the study included 19 patients with EoE and 12 patients with NOD. The mean age of the patients was 41 years for the EoE group and 30.5 years for the NOD group, with a male predominance in the EoE group (94.7% vs. 58.3%; *p* = 0.014). A significantly higher BMI was observed in the EoE group compared with in the NOD group (23.5 vs. 19.3; *p* = 0.002). No significant differences were found in smoking, alcohol use, proton pump inhibitor (PPI), nonsteroidal anti-inflammatory drug (NSAID) use, CCI, or most symptoms, with the exception of a higher incidence of food influence in the NOD group. Regarding blood test results, the EoE and NOD groups had similar serum white blood cell (WBC) counts. Regarding pathological results, the EoE group had significantly higher eosinophil, IgG4, and IgG cell counts than the NOD group. Additionally, the proportion of patients with positive TRC staining was significantly higher in the EoE group than in the NOD group (Table 1).

### 3.2. Effectiveness of Treatment Modalities for EoE and NOD Groups

PPI use was high in both groups (EoE: 94.7%, NOD: 91.7%), with no significant difference in adoption rates (*p* = 0.739). Topical steroids were only used in the EoE group (10.5%), and diet therapy was more commonly used in the EoE group (26.3%) compared with the NOD group (0%), although no statistical significance was observed (*p* = 0.056). Both groups improved after treatment; however, patients with EoE presented a significant decrease in EREFS (*p* < 0.001 before treatment vs. *p* = 0.002 after treatment). The ΔEREFS was significantly greater in patients with EoE (−1.0) compared with those with NOD (0.0), indicating greater endoscopic improvement (*p* = 0.004). Treatment response was higher in the EoE group (89.5%) than in the NOD group (66.7%). However, recurrence rates were not significantly different between the two groups (Table 2).

### 3.3. Baseline Characteristics of Endoscopic EoE Phenotypes

The study included nine patients with inflammatory, seven with fibrostenotic, and three with mixed EoE phenotypes. There were no significant differences in age, sex distribution, BMI, smoking, or NSAID use between the phenotypes. Alcohol consumption was significantly higher in the mixed phenotype group (*p* = 0.009). Endoscopic features differed markedly by phenotype: furrows were present in all inflammatory and mixed phenotype patients, rings and strictures predominated in fibrostenotic and mixed phenotypes, and edema was more common in inflammatory and mixed phenotypes. All phenotypes showed improvement in symptom scores after treatment, with no significant differences in NRS scores after treatment. The inflammatory phenotype showed the most significant reduction in EREFS after treatment, indicating a greater response to treatment compared with the other phenotypes (Table 3).

### 3.4. Serological and Pathological Differences among Endoscopic EoE Phenotypes

**Figure 5:**
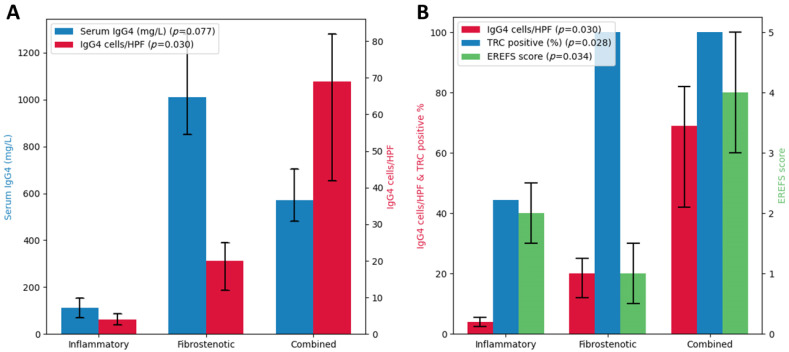
Analysis results based on EoE phenotype. (**A**) The graph provides a comparative analysis of serum IgG4 concentrations (mg/L) and IgG4 cell count per HPF across three EoE phenotypes: inflammatory, fibrostenotic, and combined. Using the analysis of variance (ANOVA) method for statistical evaluation, a discernible trend was observed in serum IgG4 levels across the phenotypes (*p* = 0.077). Moreover, a statistically significant difference was identified in IgG4 cell counts/HPF between the phenotypes (*p* = 0.030). (**B**) This graph provides IgG4 cell counts/HPF, TRC positivity rates, and pre-treatment EREFS across EoE phenotypes analyzed using ANOVA. Significant variability in IgG4 cell counts/HPF was noted across phenotypes (*p* = 0.030), with the combined phenotype showing significantly higher counts. TRC positivity rates also showed significant differences by phenotype (*p* = 0.028), with both the fibrostenotic and combined phenotypes achieving 100% positivity. Furthermore, variations in pre-treatment EREFS (*p* = 0.034) were observed, with the combined phenotype displaying higher scores, which may suggest more severe disease manifestations.

No significant differences were observed in WBC or serum eosinophil counts between the phenotypes; however, the eosinophil counts in the fibrotic phenotype were higher. Serum IgG4 levels were higher, though not statistically significant, in the fibrotic phenotype compared with the inflammatory and mixed phenotypes. IgE levels were highly variable, with the highest levels observed in the combined phenotype, although the difference was not statistically significant. Pathologically, eosinophil count per HPF was highest in the inflammatory phenotype, followed by the combined and fibrotic phenotypes, although the difference was not statistically significant. IgG4 and IgG cell counts per HPF and TRC positivity showed significant differences, with the fibrotic and combined phenotypes having higher counts and 100% TRC positivity, indicative of fibrosis (Table 4 & Figure 5).

## 4. Discussion

Recently, several studies have focused on EoE owing to its increasing prevalence [20,21]. EoE often develops at a young age, and its misdiagnosis can lead to a chronic condition [22,23]. Moreover, EoE can progress to esophageal remodeling and stricture if left untreated or become unresponsive to treatment [24,25,26]. Consequently, accurate diagnosis of EoE is important in its early stages.

Previous studies reveal discernible differences in endoscopic findings between patients with EoE and NOD, as well as the potential for endoscopic reversibility in EoE when appropriate treatment is administered [27]. Moreover, recent studies emphasize the marked histological expression of IgG4 in individuals with EoE [11,28]. Therefore, in the present study, we analyzed both endoscopic and histological features in detail to characterize IgG4 expression according to the endoscopic phenotype of EoE.

We found significant histological differences in eosinophil and IgG4 cell counts between patients with EoE and NOD, which aligns with previous findings and supports the involvement of IgG4 in EoE pathogenesis [28]. Further, to investigate the histological characteristics of different EoE phenotypes, we categorized patients with EoE into three phenotypes: inflammatory, fibrostenotic, and combined. Although there were no serological differences between the phenotypes, histological analysis showed the highest IgG4 cell counts in the combined phenotype. Notably, there was no significant difference in eosinophil cell count between the three phenotypes, emphasizing the potential significance of IgG4 rather than eosinophils in disease course and severity. Moreover, the combined phenotype also had the highest EREFS values, which is a marker of EoE severity. These observations suggest a link between IgG4 and disease severity. Although previous studies primarily focused on IgG4 expression as a diagnostic marker, it is important to consider its impact on disease severity and progression [18,29]. Therefore, future investigations should explore the variation of IgG4 expression and its potential influence on disease severity.

Additionally, we performed TRC staining to assess histological fibrosis and its association with endoscopic findings. Our results confirmed histological fibrosis in 100% of patients with ring and stricture findings in the fibrostenotic and combined phenotypes, demonstrating a strong correlation between endoscopic and histological fibrosis. Moreover, even in the inflammatory phenotype without ring and stricture findings, 44.1% of the patients exhibited histological fibrosis in the TRC staining, indicating the potential involvement of fibrosis from the early stages of EoE inflammation. This underscores the importance of histological evaluation alongside endoscopic phenotype assessment. Moreover, significant differences in ΔEREFS were observed between the EoE phenotypes, consistent with previous studies showing reversibility in the inflammatory phenotype [27]. In this study, we compared the endoscopic clinical manifestations of patients with EoE, differentiating between those with and without TRC staining. Future research focusing on correlating the severity of EoE clinical features with histological fibrosis progression, through enhanced severity scoring based on TRC staining, could underscore the significance of endoscopic histology in managing EoE. This approach aims to deepen our understanding of the clinical-histological correlation of EoE.

Our study not only confirms the diagnostic significance of IgG4 but also establishes a potential correlation between IgG4 cell count and disease severity, further indicating the importance of histological examination in EoE diagnosis. These findings highlight the need for continued research to unravel the intricate mechanisms underlying EoE and emphasize the essential role of histological assessment in complementing endoscopic phenotyping. Additionally, recent studies have emphasized the important role of IHC in diagnosing and understanding EoE, with markers such as IgG4, arachidonate-15 lipoxygenase-1 (ALOX-15), and filaggrin gaining prominence [30]. These markers not only aid in diagnosis but also help elucidate the pathogenesis of EoE to guide treatment strategies. The discovery of the effect of dupilumab, which targets IL-4 and IL-13 in the type 2 inflammatory response, is an example of how identifying specific immunological markers can advance the treatment of EoE [31,32,33]. This demonstrates the clinical importance of research to identify accurate immunologic markers for EoE.

Nonetheless, this study had some limitations. First, we reviewed all clinical data in detail; however, because this was a retrospective study, not all data were available. Second, differences between clinicians performing and interpreting the results of biopsy and endoscopy may have contributed to inter-observer variability. Third, although the prevalence of EoE is increasing in Asia, the relatively low prevalence limited the number of patients included in this study, which affected the statistical power. To address this, we recommend that future studies, including multicenter studies, should be conducted to obtain a more comprehensive picture of the relationship among histological examination, IgG4 immunostaining, and EoE. Such an approach may increase the robustness and generalizability of our findings. Finally, the timing of testing and follow-up observation did not match between the EoE and NOD groups. Despite these limitations, this is the first study in Korea to confirm IgG4 histological expression in patients with EoE.

## 5. Conclusions

The histological expression of IgG4 was higher in patients with EoE than in those with NOD and was highest in the combined phenotype, which exhibited the highest EREFS. This study reinforces the vital role of both endoscopic and histological examinations in the accurate diagnosis of EoE. Moreover, the histological variance according to EoE phenotype underscores the need for further research in this area, including prospective studies conducted at multiple medical centers. The results of this study provide insight into the development of more targeted and effective treatment strategies for patients with EoE and may help identify biomarkers for EoE that can be used for the diagnosis and monitoring of disease activity.

## Figures and Tables

**Table 1:** Baseline characteristics of patients in EoE and NOD groups.

Characteristics	EoE (*n* = 19)	NOD (*n* = 12)	*p*-Value
Age, years (IQR)	41 (18–58)	30.5 (19–46)	0.173
Male sex, *n* (%)	18 (94.7)	7 (58.3)	0.014
BMI, kg/m^2^ (IQR)	23.5 (22.0–25.5)	19.3 (18.3–21.1)	0.002
Smoking, *n* (%)	6 (31.6)	2 (16.7)	0.509
Alcohol, *n* (%)	5 (26.3)	5 (41.7)	0.484
PPI, *n* (%)	4 (21.1)	2 (16.7)	0.857
NSAIDs, *n* (%)	1 (5.3)	0 (0.0)	0.826
CCI, median (IQR)	0 (0–1)	0 (0–0)	0.326
Symptoms			
Dysphagia, *n* (%)	12 (63.2)	8 (66.7)	0.845
Heartburn, *n* (%)	4 (21.1)	2 (16.7)	0.767
Dyspepsia, *n* (%)	7 (36.8)	7 (58.3)	0.249
Reflux, *n* (%)	5 (26.3)	5 (41.7)	0.381
Food impaction, *n* (%)	0 (0.0)	2 (16.7)	0.070
Laboratory results (IQR)			
Serum WBC, count/μL	5720 (4965–6540)	7015 (4952–8620)	0.496
Pathological results (IQR)			
Eosinophil, cells/HPF	91.5 (50.0–150.0)	0.0 (0.0–0.5)	<0.001
IgG4, cells/HPF	7.5 (3.5–22.5)	0.0 (0.0–0.0)	0.003
IgG, cells/HPF	68.0 (42.0–101.0)	0.0 (0.0–0.0)	0.004
TRC positive, *n* (%)	14 (73.68)	4 (33.33)	0.029

BMI, body mass index; CCI, Charlson Comorbidity Index; EoE, eosinophilic esophagitis; HPF, high-power field; IgG, immunoglobulin; NOD, non-obstructive dysphagia; NSAID, nonsteroidal anti-inflammatory drug; PPI, proton pump inhibitor; TRC, Masson’s trichrome; WBC, white blood cell.

**Table 2:** Effectiveness of treatment modalities for patients with EoE and NOD.

	EoE (*n* = 19)	NOD (*n* = 12)	*p*-Value
Type of treatment			
PPI, *n* (%)	18 (94.7)	11 (91.7)	0.739
Topical steroid, *n* (%)	2 (10.5)	0 (0.0)	0.253
Diet therapy, *n* (%)	5 (26.3)	0 (0.0)	0.056
Pre-treatment (IQR)			
Symptoms (NRS 0–10)	3.0 (2.0–3.0)	2.0 (1.00–2.75)	0.124
EREFS score (0–9)	2.0 (1.0–3.0)	0.0 (0.0–0.0)	<0.001
Post-treatment (IQR)			
Symptoms (NRS 0–10)	0.0 (0.0–0.0)	0.0 (0.0–0.0)	0.126
EREFS score (0–9)	1.0 (0.0–2.0)	0.0 (0.0–0.0)	0.002
Response to treatment, *n* (%)	17 (89.5)	8 (66.7)	0.035
ΔEREFS score (0–9), (IQR)	−1.0 (−2.0–0.0)	0.0 (0.0–0.0)	0.004
Recurrence of symptoms, *n* (%)	4 (21.1)	1 (8.3)	0.356

EoE, eosinophilic esophagitis; EREFS, Endoscopic Reference Score; NOD, non-obstructive dysphagia; NRS, numerical rating scale; PPI, proton pump inhibitor.

**Table 3:** Baseline characteristics of patients according to EoE endoscopic phenotype.

	Inflammatory Phenotype (*n* = 9)	Fibrostenotic Phenotype (*n* = 7)	Combined Phenotype (*n* = 3)	*p*-Value
Male sex, *n* (%)	8 (88.9)	7 (100)	3 (100)	0.391
Age, years (IQR)	42.78 (29–73)	34.86 (18–44)	47.33 (41–58)	0.256
BMI, kg/m^2^ (IQR)	22.2 (20.8–23.7)	24.2 (23.4–26.0)	25.5 (23.8–27.4)	0.087
Smoking, *n* (%)	3 (33.3)	2 (28.6)	1 (33.3)	0.978
Alcohol, *n* (%)	1 (11.1)	1 (14.3)	3 (100.0)	0.009
PPI, *n* (%)	3 (33.3)	1 (14.3)	0 (0.0)	0.424
NSAIDs, *n* (%)	1 (11.1)	0 (0.0)	0 (0.0)	0.574
**Endoscopic features**				
Edema, *n* (%)	4 (44.4)	0 (0.0)	2 (66.6)	
Ring, *n* (%)	0 (0.0)	5 (71.4)	3 (100.0)	
Exudate, *n* (%)	3 (33.3)	0 (0.0)	0 (0.0)	
Furrow, *n* (%)	9 (100.0)	0 (0.0)	3 (100.0)	
Stricture, *n* (%)	0 (0.0)	2 (28.6)	0 (0.0)	
Pre-treatment EREFS (0–9), (IQR)	2.0 (2.0–3.0)	1.0 (1.0–1.0)	4.0 (3.5–5.0)	0.034
Post-treatment EREFS (0–9), (IQR)	0.5 (0.0–1.0)	1.0 (0.0–1.5)	2.5 (2.0–3.0)	
ΔEREFS (0–9), (IQR)	−2.0 (−2.0–−2.0)	−1.0 (−1.0–0.5)	−1.0 (−2.0–0.0)	0.005
**Symptoms**				
Pre-treatment NRS score (0–10), (IQR)	3.0 (3.0–4.0)	2.0 (1.5–2.5)	2.0 (1.5–3.0)	0.086
Post-treatment NRS score (0–10), (IQR)	0.0 (0.0)	0.0 (0.0)	0.0 (0.0)	0.815
Dysphagia, *n* (%)	5 (55.6)	6 (85.7%)	1 (33.3%)	0.175
Heartburn, *n* (%)	5 (55.6)	1 (14.3%)	2 (66.6%)	0.131
Reflux, *n* (%)	3 (33.3%)	1 (14.3%)	0 (0.0%)	0.125
Food impaction, *n* (%)	0 (0.0%)	0 (0.0%)	0 (0.0%)	1.000

BMI, body mass index; EoE, eosinophilic esophagitis; EREFS, Endoscopic Reference Score; NSAID, nonsteroidal anti-inflammatory drug; PPI, proton pump inhibitor.

**Table 4:** Laboratory results according to EoE endoscopic phenotype.

	Inflammatory Phenotype (*n* = 9)	Fibrostenotic Phenotype (*n* = 7)	Combined Phenotype (*n* = 3)	*p*-Value
**Blood test results (IQR)**				
Serum WBC, count/μL	5210 (4845–5965)	6330 (5390–8405)	5570 (5430–5925)	0.453
Serum eosinophil, count/μL	295.4 (191.1–374.2)	487.4 (220.0–585.0)	200.0 (144.0–600.0)	0.684
Serum eosinophil, %	5.1 (4.0–7.0)	6.8 (4.1–8.7)	3.6 (2.5–11.5)	0.666
Serum IgG4, mg/L	111.5 (69.0–154.0)	1010.0 (851.0–1280.0)	570.0 (481.0–705.0)	0.077
Serum IgG, mg/L	7313.5 (1191.1–13,436.0)	13,864.0 (11,979.0–15,367.5)	9854.0 (2482.0–12,751.0)	0.140
Serum IgE, KIU/L	42.5 (12.4–72.1)	92.0 (46.7–155.5)	204.9 (86.7–323.0)	0.915
**Pathologic results (IQR)**				
Eosinophil, cells/HPF	123.0 (86.5–150.0)	50.0 (31.0–82.0)	52.0 (51.0–101.0)	0.055
IgG4, cells/HPF	4.0 (2.5–5.5)	20.0 (12.0–25.0)	69.0 (42.0–82.0)	0.030
IgG, cells/HPF	50.0 (22.0–68.0)	89.0 (72.0–113.0)	85.0 (63.5–184.0)	0.065
TRC positive, *n* (%)	4 (44.4)	7 (100.0)	3 (100.0)	0.028

EoE, eosinophilic esophagitis; HPF, high-power field; TRC, Masson’s trichrome; WBC, white blood cell.

## Data Availability

All data used in this study are available in this article.
